# False lumen embolization and false lumen stent-graft techniques for ruptured post-dissection thoracoabdominal aneurysm

**DOI:** 10.1093/jscr/rjae478

**Published:** 2024-08-05

**Authors:** Takumi Umibe, Hironobu Nishiori, Hiroki Ikeuchi, Shintaroh Koizumi, Hideki Ueda, Goro Matsumiya

**Affiliations:** Department of Cardiovascular Surgery, Chiba University Hospital, 1-8-1, Inohana, Chuo-Ku, Chiba 286-0041, Japan; Department of Cardiovascular Surgery, Chiba University Hospital, 1-8-1, Inohana, Chuo-Ku, Chiba 286-0041, Japan; Department of Cardiovascular Surgery, Chiba University Hospital, 1-8-1, Inohana, Chuo-Ku, Chiba 286-0041, Japan; Department of Cardiovascular Surgery, Chiba University Hospital, 1-8-1, Inohana, Chuo-Ku, Chiba 286-0041, Japan; Minami-Senju Hospital, Division of Cardiology, Tokyo, Japan; Department of Cardiovascular Surgery, Chiba University Hospital, 1-8-1, Inohana, Chuo-Ku, Chiba 286-0041, Japan

**Keywords:** false lumen stent graft, Candy-plug, post-dissection ruptured aortic aneurysm

## Abstract

This report details the successful endovascular repair of a ruptured thoracoabdominal aortic aneurysm in a patient with chronic type B aortic dissection. The procedure consisted of thoracic endovascular aortic repair, abdominal endovascular aortic repair, false lumen (FL) embolization with Candy-Plug, and FL stent-graft technique. The approach effectively regulated FL inflow, achieving complete FL closure. The patient was discharged without major complications including spinal cord ischemia or renal failure, and the long-term outcome is also favorable with reduction of the aneurysm size. The follow-up results have shown a reduction in the aneurysm size. This less invasive method could be an option of treatments for post-dissection thoracoabdominal aortic aneurysms, especially in ruptured cases.

## Introduction

Chronic type B aortic dissection (CTBAD) with patent false lumen (FL) often presents with FL dilation and rupture due to residual re-entry tears. Open repair for CTBAD carries various risks, including spinal cord ischemia (SCI), and renal failure [1]. Here, we report a case of ruptured TAAA successfully repaired using endovascular repair combined with FL embolization and FL stent-graft technique. With this approach, we could save the patient’s life, and reduce the aneurysmal size throughout the thoracoabdominal region significantly in the long term.

## Case report

**Figure 1:**
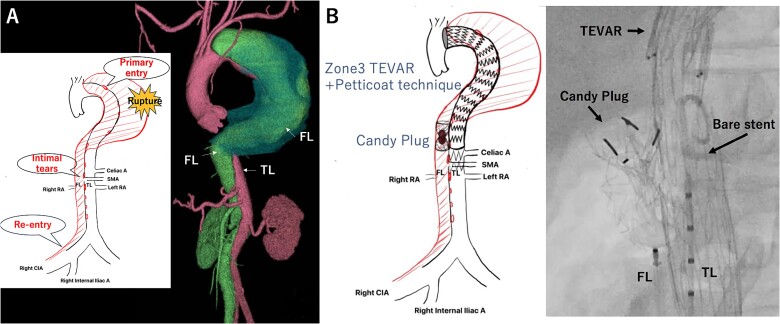
Preoperative computed tomography 3D reconstruction showing the FL extended from the descending aorta to the right common iliac artery. The schema showing multiple intimal tears at thoracic and abdominal levels (A). Intraoperative angiography showing Candy plug made with Excluder aortic extender and AMPLATZER Vascular Plug II deployed in the FL at the level of bare stent of Zenith TX-D in the TL (B). TL: true lumen FL: false lumen RA: renal artery CA: celiac artery SMA: superior mesenteric artery TEVAR: thoracic endovascular aortic repair.

A 57-year-old man, with a history of acute Stanford type B aortic dissection from the descending thoracic aorta extending to the right common iliac artery (CIA), had been treated conservatively for 10 years. He admitted to the hospital with chief complaint of sudden-onset back pain. The contrast-enhanced computed tomography (CT) imaging revealed CTBAD extending from the distal aortic arch to the right CIA, with a rupture in the thoracic aorta. The diameter of TAAA was 71 mm. The primary entry was located at the descending aorta close to the subclavian artery. Multiple intimal tears were present at the descending aorta, as well as at the level of superior mesenteric artery (SMA) and renal artery (RA). The right RA was originated from the FL (Fig. 1A). Considering the invasiveness of conventional aortic replacement under left thoracotomy, we decided to perform endovascular repair.

The bilateral femoral arteries were approached under general anesthesia. First, we completed TEVAR with Zenith TX-D (Cook Medical Incorporated, Bloomington, IN). The proximal covered stent was deployed just below the subclavian artery landing to the Th11 level of descending aorta. The extension bare stent was deployed just above the left RA. Then we approached to the FL through the re-entry tear in right CIA, and we occluded the FL at the SMA level with modified Candy-Plug technique using Excluder aortic extender (WL Gore & Associates, Newark, DE) and AMPLATZER Vascular Plug II (Abbott Laboratories, Chicago, IL) [2] (Fig. 1B). The aortography confirmed that blood flow into the FL at the thoracic level was well regulated.

However, on the third postoperative day, a contrast-enhanced CT showed re-rupture of the TAAA due to the residual reversal flow into the FL through the Candy-Plug. Therefore, we decided to perform additional endovascular repair.

**Figure 2:**
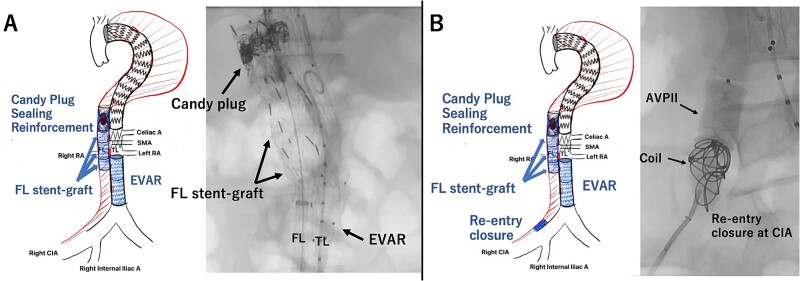
Intraoperative angiography showing the Excluder deployed in the FL closing intimal tears neat the renal artery (A). The re-entry at the CIA was closed with AMPATZER Vascular Plug II and Interlock microcoils (B). TL: true lumen FL: false lumen RA: renal artery CA: celiac artery SMA: superior mesenteric artery CIA: common iliac artery.

**Figure 3:**
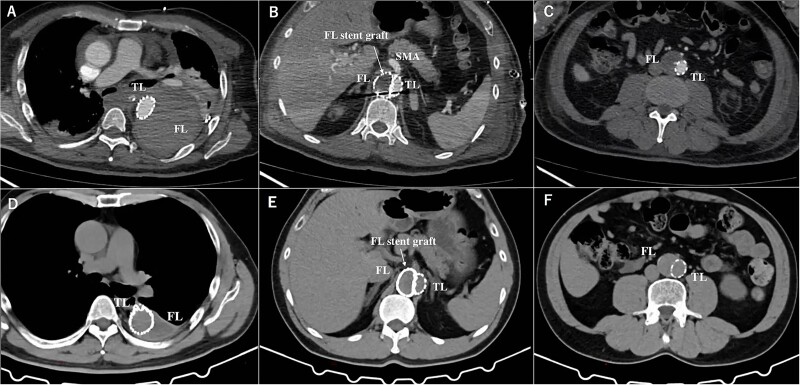
The postoperative contrasted computed tomography at thoracic level (A), at SMA level (B), at abdominal level (C). The computed tomography three years after the operation at thoracic level (D), at SMA level (E), at abdominal level (F). TL: true lumen FL: false lumen RA: renal artery CA: celiac artery SMA: superior mesenteric artery CIA: common iliac artery.

First, the gutter of the Candy-Plug was occluded using AMPLATZER Vascular Plug IV (Abbott Laboratories, Chicago, IL) and Interlock™ detachable microcoils (Boston Scientific, Marlborough, MA). Then, the Endurant II Iliac Extension (Medtronic, Dublin, Ireland) was deployed from just below the left RA to the aortic bifurcation closing the infra-renal abdominal intimal tears. A few residual intimal tears were identified at the visceral segment, including the punch-out lesion of the right RA. These intimal tears were closed using three pieces of Excluder aortic extender (WL Gore & Associates, Newark, DE) from the FL side sacrificing the right RA intentionally. This FL stent-graft was landed in the FL at the level of the distal end of Zenith TX-D proximally and at the level of the proximal end of the Endurant II iliac Extension distally to avoid excessive force to the intimal flap (Fig. 2A). Finally, a AMPLATZER Vascular Plug II (Abbott Laboratories, Chicago, IL) and Interlock™ were placed in the FL of right CIA to close the re-entry tear at right CIA (Fig. 2B). The postoperative CT imaging showed complete thrombosis of the FL, and he discharged on postoperative day 24 (Fig. 3). Two years after the operation, the CT imaging showed decreased aortic diameter throughout thoracoabdominal region with a thoracic aortic diameter of 51 mm.

## Discussion

Open surgery for CTBAD carries risks including SCI, visceral and limb ischemia, and renal failure [1]. In ruptured cases, the anticipated surgical risk is presumed to be even greater [3]. In this case, the aortic dissection extended just below the left subclavian artery, making traditional open surgical repair which require open proximal anastomosis under deep hypothermic circulatory arrest, highly invasive and associated with significant surgical risk. In recent years, endovascular strategies including FL embolization with Candy-plug, have been reported as good treatment options for FL dilation of CTBAD [4]. In this case, after deploying TEVAR, Candy-Plug was placed in the FL to prevent blood flow into the FL at the thoracic level. Utilizing the Petticoat technique, a bare stent was positioned on the true lumen side, providing support for the safe placement of the Candy-Plug in the FL. Furthermore, to completely eliminate FL flow, closure of all re-entry tears was achieved through treatment involving EVAR and the FL stent-graft technique.

The use of FL stent-grafts is highly beneficial for closing intimal tears in the visceral segment area; however, there are anatomical constraints to consider [5]. The origin of abdominal branches from the FL must be assessed. If any abdominal branches originate from the FL, they would be occluded when placing FL stent-grafts. In this emergency case, from a technical standpoint, it is possible to use a covered stent to bridge from the right RA to true lumen through the intimal tear in the flap, securing blood flow to the right RA before deploying FL stent-grafts. However, if residual flow into the FL remains through the gap between the intimal tear and the covered stent, additional treatments would become quite challenging.

The size and choice of devices is another point. The size of the FL stent-graft is chosen to be 5%–10% oversized compared to the calculated diameter based on the FL circumference length [6]. To avoid potential damage to the flap, we put for slightly oversized stent-graft. The Excluder Aortic Extender is our preferred choice for FL stent-graft, being the most readily available in our hospital inventory for emergency use.

The combination of TEVAR, EVAR, Candy-Plug, and FL stent-graft can be useful technique for completely occluding intimal tears, including at the visceral segment, and control the reversal blood flow into the FL. This approach can be a treatment option for post-dissection TAAA in ruptured cases.

## Data Availability

Not applicable.
